# The critical omission of CRRT dose in comparative studies of RRT modalities for sepsis-associated AKI

**DOI:** 10.1186/s40981-025-00794-9

**Published:** 2025-08-09

**Authors:** Minmin Wang, Qiang Yao

**Affiliations:** Department of Medical Affairs, Vantive Health LLC, Shanghai, China

To the Editor,

We read with interest the recent study by Okano et al. comparing intermittent renal replacement therapy (IRRT) and continuous renal replacement therapy (CRRT) in sepsis-associated AKI (S-AKI) (JA Clin Rep. 2025; 11:24) [1]. While the authors provide valuable insights into the comparable mortality outcomes between IRRT and CRRT, a critical limitation undermines the interpretation of their findings: the absence of data on CRRT dosing.

CRRT dose is a well-established determinant of outcomes in critically ill patients. The KDIGO AKI guidelines recommend a standard dose of 20–25 ml/kg/h [2]. Notably, in practice, the average CRRT dose in Japan is 10–15 ml/kg/h, as mentioned in several Japanese guidelines [3, 4]. While observational studies suggest equivalent outcomes with lower doses [5, 6], their generalizability to S-AKI remains unproven.

Okano et al. did not report CRRT dosing parameters (e.g., effluent rate, downtime), rendering it impossible to assess whether delivered doses aligned with Japanese standards or international recommendations. This omission is critical for two reasons: First, suboptimal CRRT dosing may confound mortality outcomes. If Japanese CRRT doses were uniformly low, the observed lack of difference between CRRT and IRRT could reflect underdosing rather than true equivalence. Second, prior trials comparing IRRT and CRRT emphasized dose parity between modalities (i.e., ensuring equivalent total solute clearance when comparing outcomes) [7], a factor not addressed in this study.

The authors also overlooked the interplay between dose and hemodynamic stability. CRRT is often preferred for hemodynamically unstable patients due to its gradual fluid and solute removal, which theoretically minimizes hemodynamic fluctuations. However, lower CRRT doses may compromise solute clearance, potentially offsetting its benefits in sepsis. For instance, inadequate clearance of inflammatory mediators or toxins in S-AKI could worsen outcomes, even if hemodynamic stability is maintained. Conversely, IRRT’s higher intensity might compensate for shorter treatment durations but risks hemodynamic instability during rapid solute shifts. Without dose data, the study cannot disentangle whether outcomes reflect modality efficacy, dosing adequacy, or the balance between solute control and hemodynamic tolerance.

Furthermore, while Japanese studies propose that lower CRRT doses yield outcomes comparable to higher doses, these findings are not definitive. The lack of RCTs specifically comparing low-dose CRRT (as practiced in Japan) with guideline-recommended doses in S-AKI cohorts leaves a critical evidence gap. For example, Uchino et al.’s observational study found no mortality difference between Japanese low-dose CRRT and historical international standards, but confounding factors such as patient selection and concurrent therapies cannot be ruled out [8]. Until RCTs validate these findings, the safety and efficacy of low-dose CRRT in S-AKI remain uncertain.

We urge future studies to address these gaps by rigorously reporting CRRT parameters (effluent rate, downtime) and stratifying analyses by dose thresholds. Additionally, comparing Japanese low-dose CRRT with guideline-recommended doses in S-AKI cohorts through RCTs could clarify whether current practices are evidence-based or necessitate recalibration. Until then, conclusions about CRRT and IRRT equivalence in S-AKI remain provisional, as unmeasured dose variability may mask true differences in efficacy.

## Data Availability

No datasets were generated or analyzed during the current study.

## Abbreviations

RRT: Renal replacement therapy
IRRT: Intermittent renal replacement therapy
CRRT: Continuous renal replacement therapy
S-AKI: Sepsis-associated AKI
RCTs: Randomized controlled trials
